# Taxonomic status and molecular phylogeography of two sibling species of *Polytremis* (Lepidoptera: Hesperiidae)

**DOI:** 10.1038/srep20820

**Published:** 2016-02-10

**Authors:** Weibin Jiang, Haiyan He, Yingdong Li, Mengyi Ren, Yazhong Ma, Lingli Zheng, Jianqing Zhu, Weidong Yu

**Affiliations:** 1 Shanghai Normal University, College of Life and Environmental Sciences, Shanghai, People’s Republic of China; 2 Shanghai Zoological Park, Shanghai, People’s Republic of China

## Abstract

The skipper *Polytremis theca* species complex is widely distributed in the south of the Qinling Mountains in China. A recent study of the *Polytremis* genus suggested that this species might encompass two differentiated lineages. We tested this hypothesis, by carrying out a phylogenetic study of this agricultural pest based on nationwide sampling and the evaluation of mitochondrial and nuclear DNA markers. We show that this species is actually an amalgamation of two sibling taxa (*P. t. theca* and *P. t. fukia*), which displayed levels of genetic divergence as great as those generally found between sister species in the *Polytremis* genus, suggesting that they actually correspond to two distinct species. The Divergence time estimates suggest that an active period of speciation within *Polytremis* occurred within the Pleistocene eras. Based on its distinct phylogenetic placement and geographical isolation, we suggest that the subspecies should be elevated to full species status under the phylogenetic species concept, which has significant management implications.

Evans^1^ described a lot of skipper species from Europe, Asia and Australia. He often described varieties, now considered subspecies under the current Code of Zoological Nomenclature^2^, when specimens exhibited morphology similar to known taxa. It appears to follow the classical typological species concept where small differences represented regional variation in the species. Close inspection of such intraspecific variations has sometimes revealed that the species’ actually contain several closely related multiple species, so-called ‘sibling species’ or ‘cryptic species’, that are difficult to distinguish morphologically from each other, at least without skilful eyes^3^,^4^,^5^. With the development of molecular techniques and the collection of greater number of specimens, some of his subspecies were argued, revised and elevated to species rank. Chiba & Eliot^6^, Devyatkin & Monastyrskii^7^, Guo *et al*.^8^ reevaluated *Parnara guttatus batta* Evans, 1949 by morphological features, molecular data and biogeographical implications and treated it as an independent species named *Parnara batta* Evans, 1949. Chiba *et al*.^9^ and Hsu^10^ regarded *Polytremis menica kirazana* Evans, 1949 as valid species-level named *Polytremis kiraizana* Sonan, 1938 by further morphological and phylogenetic analysis.

The skipper *Polytremis theca* species complex is widely distributed in the south of the Qinling Mountains in China, except Hainan Province and the southern tropical regions of Yunnan Province^1^,^11^,^12^. Evans^1^ described *P. t. theca* Evans, 1937 from Sichuan Province. He then described the *P. t. fukia* Evans, 1940 from Fujian Province that was lighter in coloration than the typical *P. theca* and existed 1or 2 white spots in space Cu_2_ on the upperside of forewing*. P. t. theca* and *P. t. fukia* are nearly separated geographically.

If genetic differentiation has occurred, the taxonomic status of *P. theca* species complex can be reevaluation by molecular techniques. In a preliminary study of molecular phylogeny of the genus *Polytremis* Mabille 1904 using mitochondrial cytochrome c oxidase I (COI), we found the inter-subspecific distance between *P. t. theca* and *P. t. fukia* ranged up to 4.2%, which is higher than some inter-specific genetic distances in *Polytremis*^13^. Additionally, *P. theca* is also the only species whose intra-specific distance is more than 3%, a thresholds of species identification have been proposed in Lepidoptera for COI, in genus *Polytremis*^14^. Thus, we suspected the possible existence of a sibling species paired or the cryptic diversity in the species. However, this assertion was based on a very small number of samples and required confirmation in a larger sample. To answer the question, we conducted molecular phylogenetic analyses of *P. theca* species complex collected from 13 geographic localities in China using a region of mitochondrial genes (COI) and a region of nuclear genes (*wingless*).

## Materials and methods

### Samples collection

**Figure 1:**
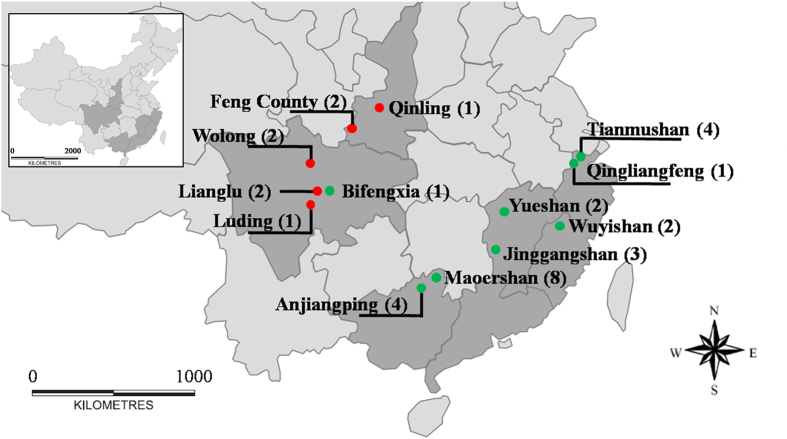
Distribution of the specimens of *P. theca* collected in China; red circle, *P. t. theca*, green circle, *P. t. fukia.* We modified the map from Jiang *et al*.^13^

We collected specimens of *P. t. theca* (8) from five local regions and *P. t. fukia* (25) from eight different localities in China from 2008 to 2014 (Fig. 1; Table 1), and preliminarily identified them based on traditional wing and genitalia morphology characters recommended by Evans^1^, Eliot^15^ and Chou^16^. Our sampling of 33 specimens covered the major range of the *P. theca* (Fig. 1). The outgroups included eight specimens of *P. nascens* Leech, 1893, five specimens of *P. mencia* Moore, 1877 (Table 1). All specimens were caught in the field, preserved by dehydration in small envelopes and dried with silica desiccant for further processing.

### DNA extraction and sequencing

The DNA was isolated from legs of adult butterfly using a QIAamp DNA Mini kit (Qiagen, Hilden, Germany) essentially following the manufacturer’s instructions but with some modification. Briefly, after adding proteinase K and buffer AL (QIAGEN^®^), the mixed homogeneous solution was incubated at 70 °C for 2 h. Subsequently, 200 μL of 100% ethanol was added and the mixture transferred to a QIAamp spin column. The mixture in the spin column was subjected to 3cycles of centrifugation at full speed (14,000*g*) for 1 min and the filtrate was returned to the spin column to increase the amount of DNA obtained. Partial sequences of mitochondrial gene COI (487 bp) was amplified and sequenced with primers HCO2198: 5′-TAA ACT TCA GGG TGA CCA AAA AAT CA-3′ and LCO1490: 5′-GGT CAA CAA ATC ATA AAG ATA TTG G-3′^17^. The nuclear DNA fragments of *wingless* (389) were amplified and sequenced with primers WG1z: 5′-GAR TGY AAR TGY CAY GG-3′ and WG2a: 5′-ACT ICG CAR CAC CAR TGG AAT GTR CA-3′^18^. The PCR for all amplicons were conducted in 20 μL final volume reactions containing 1 μL template DNA, 0.4 μM each of the amplification primers, 200 μM each dNTP, 1.5 mM MgCl_2_, 50 mM KCl, 10 mM Tris–HCl (pH 8.3) and 2 U Taq polymerase (Takara, Otsu, Shiga, Japan). The thermal profile used was as follows. COI: 95 °C denaturation for 5 min followed by 35 cycles of 94 °C for 45 s, 42 °C for 1 min, 90 °C for 90 s and a final elongation step at 72 °C for 10 min. *Wingless*: 95 °C denaturation for 5 min followed by 30 cycles of 94 °C for 60 s, annealing at 55 °C for 60 s and extension at 72 °C for 2 min, with a final extension of 72 °C for 10 min.

Extraction blanks were run in all reactions to control for contamination during the extraction and PCR processes. The amplification products were subjected to electrophoresis in a 2% (w/v) agarose gel in TAE buffer (0.04 M Tris–acetate, 0.001 M EDTA) with a DL1000 ladder size marker (Takara, Otsu, Shiga, Japan) to determine whether the amplification reactions were successful. In addition, after electrophoresis in agarose gels, the amplification products were extracted using the Wizard SV Gel and PCR Clean-up System (Promega, Madison, WI, USA) for sequencing. Finally, all the haplotypes obtained were deposited in GenBank (Table 1).

### Statistical analyses

The sequence data of the mitochondrial COI and nuclear *wingless* were aligned with published homologous sequence from genus *Polytremis* (e. g., *P. eltola* Hewitson, 1869, *P. discreta* Elwes & Edwards, 1897, etc.) as well as *Borbo cinnara* Wallace, 1866 and *Pseudoborbo bevani* Moore, 1878 (presented in Table 1) respectively, translated to amino acid sequences to check for nuclear mitochondrial pseudogenes (numts) and pruned to remove redundant sequences with Bioedit v.7.0^19^. The haplotype sequence matrix was used for all subsequent phylogenetic analyses (Table 1). Phylogenetic trees were constructed by the ML (maximum-likelihood) methods with PhyML^20^. Modeltest 3.7^21^ was used to select the optimal nucleotide substitution models following the Akaike Information Criterion (AIC). In the ML analysis, a heuristic search was conducted. The starting tree for branch-swapping was from stepwise addition. Nodal support of the ML tree was estimated by 1000 bootstraps. Network profile of the haplotypes identified in *P. t. theca, P. t. fukia, P. nascens* and *P. mencia* was constructed with Network4.5 using the median-joining method^22^. The haplotype diversity (Hd) and nucleotide diversity (π) for *P. t. theca* and *P. t. fukia* were estimated by DnaSP4.90^23^.

For the COI data set, pairwise F_ST_ was also calculated with Arlequin v3.0^24^, which accurately reflects patterns of genetic variation. Correspondingly, gene flow was estimated in Arlequin v3.0^24^. Two level hierarchical analyses of molecular variance (AMOVA) were conducted to evaluate possible population genetic structure of *P. t. theca* and *P. t. fukia* using Arlequin v3.0 with 1,000 permutations. We calculated Tajima’s D^25^ and Fu’s F statistic^26^ and ran 10,000 coalescent simulations for each statistic to create 95% confidence intervals investigate the historical population demographics and testing whether the sequences conformed to the expectations of neutrality. Pairwise mismatch distribution analyses were performed for all *P. theca* specimens and two subspeices specimens separately to find the evidence of past demographic expansions using DnaSP4.90^23^. The times to the most recent common ancestor of the major lineages and the whole population were estimated using relax-clock molecular dating estimation implemented in the BEAST 1.5.2^27^. Analyses using the HKY model of nucleotide substitution with gamma distributed rate variation among sites were performed. The Yule speciation method was assumed and the nucleotide substitution rate of 3.54% per million years that has generally been calibrated for COI in insect^28^ was used. Chains were run for 50 million generations, with the first 20% discarded as burn-in. The results were summarized through TRACER 1.5^29^.

## Result

### Genetic Divergences and haplotype networks

**Figure 2:**
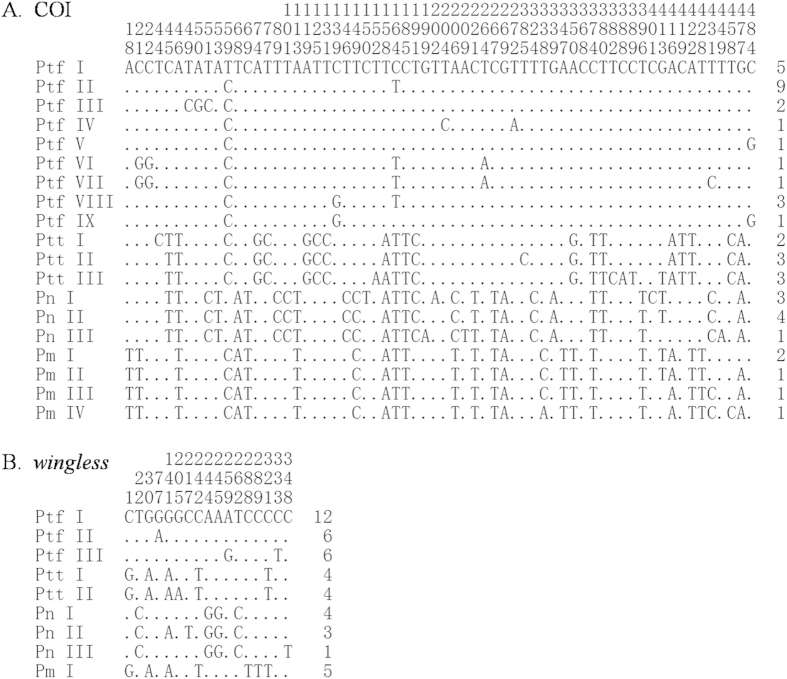
Sequence nucleotide substitutions of (**A**) the 19 haplotypes identified in 45 COI gene sequences and (**B**) the 9 haplotypes identified in 45 *wingless* gene sequences of *P. t. theca* (Ptt), *P. t. fukia* (Ptf), *P. nascens* (Pn) and *P. mencia* (Pm). The numbers on top of sequences indicates the nucleotide position which is relative to beginning of the fragment investigated in this study. The last column shows the number of individuals shared each haplotype. Dots (·) denote the identity with the reference sequence (Ptf I).

**Figure 3:**
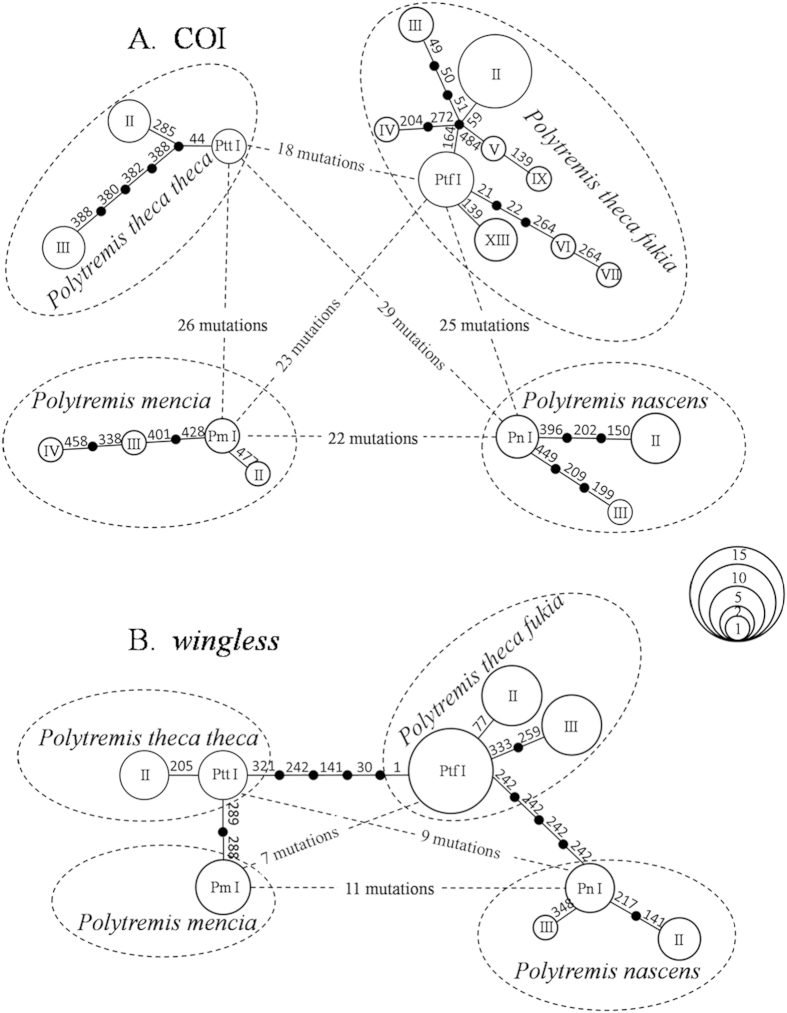
Network profile of (**A**) COI and (**B**) *wingless* gene haplotypes based on the nucleotide sequences of *P. t. theca, P. t. fukia, P. nascens* and *P. mencia*. Each haplotype is represented by a circle. The area of the circles is proportional to the number of individuals that shared the haplotype. The circles labelled 1, 2, 5, 10 and 15 on the right hand side represent the number of individuals. The number of unique and shared haplotypes identified in different locations is shown in Table 1. The order of mutations (which were scored relative to haplotype Ptt I) on a branch is arbitrary.

All 46 samples yielded high-quality of DNA and were successfully sequenced for the mitochondrial COI and nuclear *wingless* (accession numbers KR911919-KR911947, Table 1, Fig. 2). The data set of COI alignment contains 487 nucleotide positions, of which 64 positions are variable and 47 are parsimony informative. The mean base composition of the fragment shows a strong bias of A + T (T 39.4%, C 18.2%, A 28.7% and G 13.7%), as found commonly in insect mitochondrial genomes^30^. Nineteen haplotypes were identified in all 46 samples (three in *P. t. theca*, nine in *P. t. fukia*, three in *P. nascens* and four in *P. mencia*) and the haplotype network was constructed and presented in Fig. 3. There was no shared haplotype among the four taxa. Haplotypes of the same taxon differed from each other by no more than five mutation distance. The five mutation distance existed between the haplotype Ptt I and Ptt III of *P. t. theca*. The potential ancestral haplotype of *P. t. fukia*, defined by its central position in the network, was designated as Ptf I, which was found in three samples from West Tianmu Mountains, one from Jinggang Mountains, and one from Lingui. Ptf II was the most common haplotype in *P. t. fukia* and shared with ten samples. Haplotype Ptf III was found in two samples from Wuyishan. Haplotype Ptf XIII was identified in two sampled from Maoershan and one sampled from Anjiangpin. The remaining haplotypes of *P. t. fukia* occurred in only one individual. At least 18 nucleotide substitutions were observed between the potential ancestral haplotypes of *P. t. theca* (Ptt I) and that of *P. t. fukia* (Ptf I). In addition, the haplotype (Pn I) of *P. nascens* differed from Ptt I and Ptf I by 29 and 25 nucleotide substitutions while the haplotype (Pm I) of *P. mencia* differed from Ptt I and Ptf I by 26 and 23 nucleotide substitutions, respectively (Figs 2 and 3).

The data set of nuclear *wingless* contains 390 nucleotide positions without gaps or stop codons, of which 18 positions are variable and 9 are parsimony informative. In total, ten haplotypes were found in all samples, in which two haplotypes were found in *P. t. theca*, four in *P. t. fukia*, three in *P. nascens* and one in *P. mencia*. All the haplotypes were used for network construction with the software Network 4.5 using the median-joining method (Fig. 3). Haplotypes of the same taxon differed from each other by no more than two mutation distance, in particular, the two haplotypes in 13 samples of *P. t. theca* differed by only one-mutation distance. Five nucleotide substitutions were observed between the potential ancestral haplotypes of *P. t. theca* (Ptt I) and that of *P. t. fukia* (Ptf I). In addition, the haplotype (Pn I) of *P. nascens* differed from Ptt I and Ptf I by nine and five nucleotide substitutions while the haplotype (Pm I) of *P. mencia* differed from Ptt I and Ptf I by two and seven nucleotide substitutions, respectively (Figs 2 and 3).

Overall, *P. t. theca* had a lower diversity than *P. t. fukia* according to the result of analysis of both mitochondrial COI and nuclear *wingless*. The haplotype diversity (Hd) and nucleotide diversity (π) for *P. t. theca* and *P. t. fukia* are given in Table 2. Additionally, they differed from each other by 5.07 ± 0.49% (4.3–5.9% divergence) for the COI sequences and by 1.70 ± 0.27% (1.3–2.1% divergence) for the *wingless* sequences.

### Phylogenetic and population structure analysis

**Figure 4:**
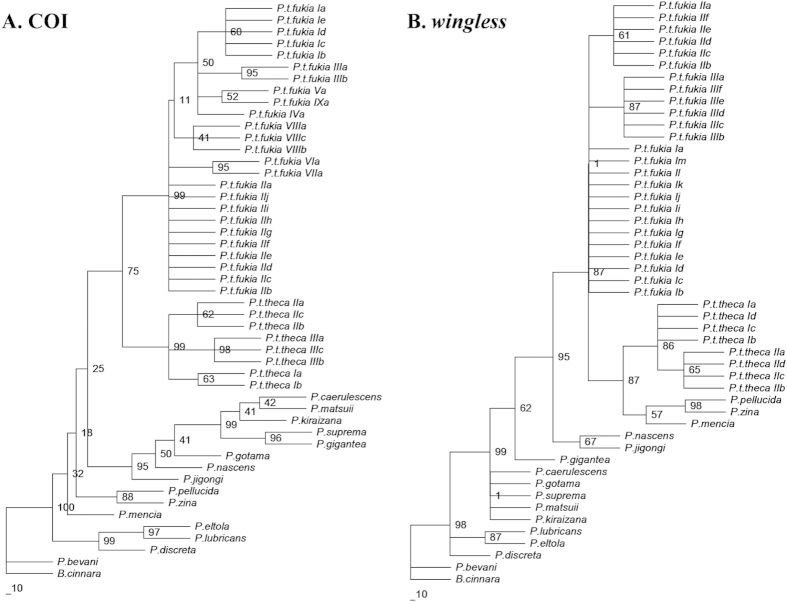
The maximum-likelihood tree for mitochondrial COI and nuclear *wingless* haplotypes of *Polytremis*. (**A**) The submodel GTR + I + G was selected for the COI gene sequence data; (**B**) The submodel GTR + G was selected for the *wingless* sequence data.

Phylogenetic analyses were performed using ML method. For the COI gene sequence data, the submodel GTR + I + G was selected. For the *wingless* sequence data, the submodel GTR + G was selected. Figure 4A shows the ML tree based on the data set of COI. On the phylogeny, the haplotypes of *P. theca* were split into two discrete clades. One clade with three haplotypes consisted exclusively of the *P. t. theca* (strongly supported by the bootstrap probability of 99%), whereas the other clade with nine haplotypes exclusively consisted of the *P. t. fukia* (strongly supported with bootstrap probability of 99%). Figure 4B shows the ML tree based on the data set of *wingless*. Three haplotypes, constituted a monophyletic group, exclusively containing *P. t. fukia* butterflies whereas two haplotypes of the *P. t. theca, P. pellucida* Murray, 1875, *P. zina* Evans, 1932 and *P. mencia* Moore, 1877 constituted a strongly supported clade (89%). On the other hand, two *P. t. theca* haplotypes formed a strongly supported clade (86%). Mitochondrial COI and nuclear *wingless* strongly supports the distinction between *P. t. theca* and *P. t. fukia.*

The AMOVA for the COI sequences of *P. t. theca* and *P. t. fukia* revealed that 88.53% of the genetic variation was among populations and 11.47% was within populations (Table 3). The average ΦST value as 0.896 (p < 0.01), suggesting significant genetic variation among the populations. Pairwise estimates of F_ST_ (0.885) and gene flow (Nm = 0.065) between *P. t. theca* and *P. t. fukia* suggests that the subspecies in this species are highly differentiated.

### Demographic inference and estimation of divergence times

**Figure 5:**
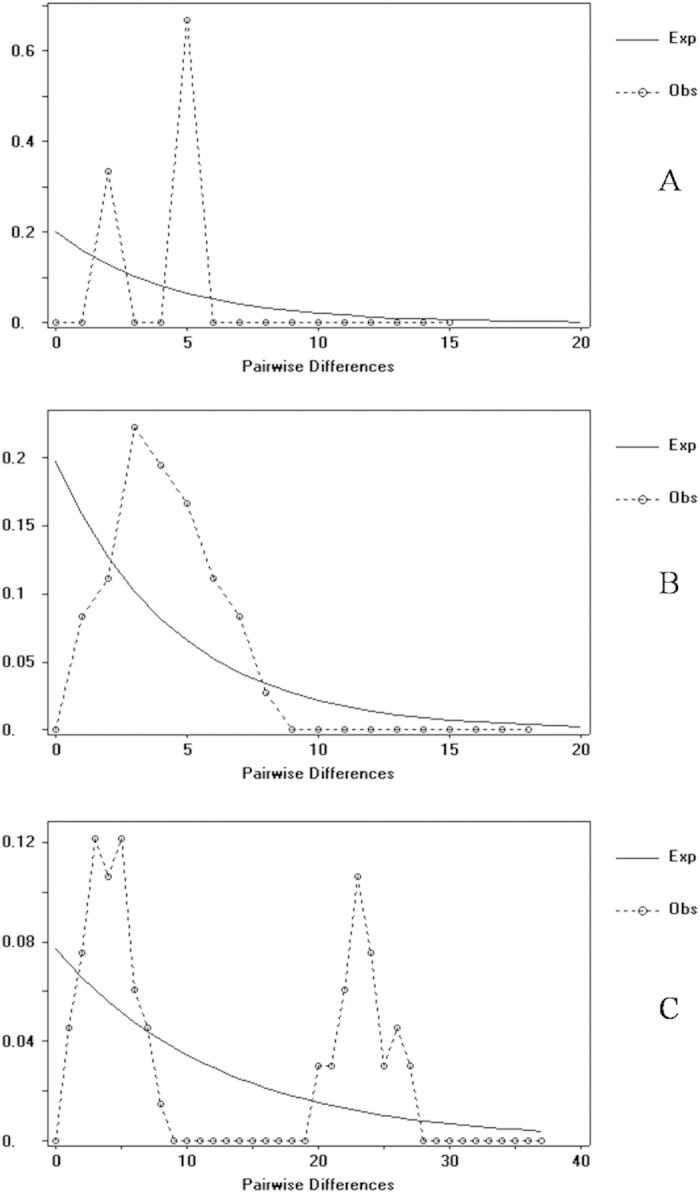
Mismatch distributions of pairwise nucleotide differences for (**A**) *P. t. theca*, (**B**) *P. t. fukia* and (**C**) *P. t. theca* and *P. t. fukia*. X axis: Pairwise Differences. Y axis: Frequency. The circles show the observed distribution of pairwise difference. The solid lines represent the expected equilibruim distributions.

**Figure 6:**
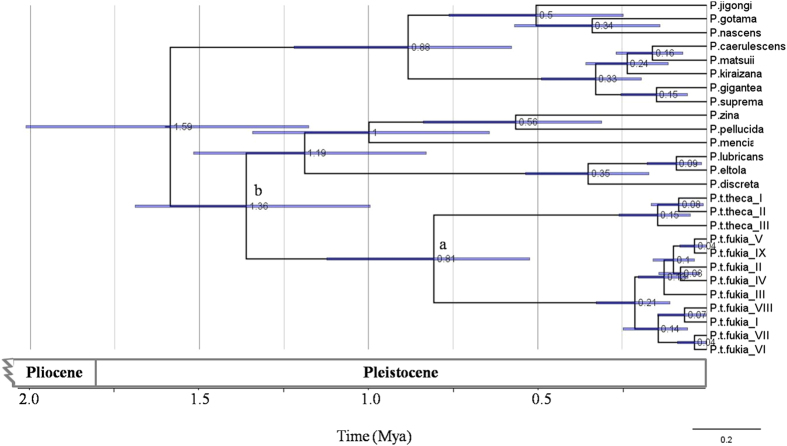
Bayesian Inference (BI) tree of mtDNA datasets for *Polytremis* using uncorrelated lognormal relaxed clock in BEAST v1.5.2, shows estimates of time since the most recent common ancestor for each major node and main mitochondrial clades. Posterior probabilities of nodes are shown below node branch when higher than 0.95. The violet bars indicate 95% highest posterior density interval (HPD) of the node ages.

Demographic history changes were analyzed for *P. t. theca* and *P. t. fukia* populations through neutrality tests and mismatch distribution. The neutrality tests reveal that the Mitochondrial COI appear to be not evolving neutrally as Fu’s F values in *P. t. fukia* group are negative significantly (Table 2). The Tajima’s D and Fu’s F values were non-significantly positive in *P. t. theca* group and all *P. theca* samples group. The mismatch analysis yielded a unimodal distribution of pairwise differences for *P. t. fukia* (Fig. 5B) compared to the multimodal distribution of *P. t. theca* samples (Fig. 5A) and the pooled samples (Fig. 5C). According to Rogers and Harpending^31^, the observed curves with unimodal representing population expansion and the observed curves with many peaks or resemblance to expected curves mean equilibrium population, which further elucidating the demographic history of *P. theca*. The results suggest that population expansion in *P. t. fukia* and population equilibrium in *P. t. theca.* Divergence time analysis with an uncorrelated lognormal relaxed clock run in BEAST produced a tree with a topology similar to ML tree (Fig. 6). *P. t. theca* diverged from *P. t. fukia* around 0.81 (HPD = 0.53–1.28) million years ago (Mya) during the Pleistocene (node a in Fig. 6). *P. theca* diverged from other congeners included in the analysis about 1.36 (HPD = 1.02–1.53) Mya during the Pleistocene eras (node b in Fig. 6).

## Discussion

There is a small region of overlap in central Sichuan province in the distribution of *P. t. theca* and *P. t. fukia*, but otherwise they are not sympatric (Fig. 1). *P. t. theca* inhabits the higher elevations of west-central Sichuang Province and the Qinling Mountains^16^. *P. t. fukia* occurs in the whole southeastern of China, from northern Zhejiang Province, Jiangxi Province to east-central Yunnan Province^9^,^32^,^33^. According to the description on morphological variation between *P. t. theca* and *P. t. fukia*^12^, we found a different morphological feature existing in female genitalia except for the different color and spot number in some part of wings (Table 4). The ductus bursae of female *P. t. theca* is thinner than that of female *P. t. fukia*. We suspected thetaxonomic status of the subspecies from their geographic separation and the morphological variation.

Mitochondrial haplotypes sampled from *P. theca* form well supported clades that closely correspond with subspecific boundaries delimited primarily on the basis of wing color and pattern (Fig. 4A). The haplotype clades associated with both subspecies are deeply genetically divergent, differing from each other by 5.07 ± 0.49% (4.3–5.9% divergence). This degree of divergence suggests that evolutionary separation of both subspecies occurred about 0.81 (HPD = 0.53–1.28) Mya, likely sometime during the Pleistocene based on a molecular clock calibration of 3.54% pairwise divergence per million years for a homologous mtDNA fragment in other insect species^28^. (fossil data and geographical calibration event are not available for this group). It is noteworthy for the nuclear *wingless* sequences that *P. t. theca* and *P. mencia* are considered distinct species with a genetic divergence of 0.65 ± 0.15% while the *P. t. fukia* is currently considered a subspecies of the *P. t. theca* despite 1.70 ± 0.27% sequence divergence. The phylogenetic of *wingless* gene indicates that the *P. theca* is paraphyly with three species sister to the clade of *P. t. theca* (Fig. 4B). While 100% of *P. t. fukia* constitutes one separate clade, the clade consisting of *P. t. theca* also includes *P. pellucid, P. zina* and *P. mencia*. The clade consisting of *P. t. theca* is not monophyletic, but complex. This suggests that *P. t. theca* and *P. t. fukia* differ from each other, as evident from the COI tree where they form two separate clades (Fig. 4A). Concordance between strongly differentiated mtDNA, nuclear haplotype clades and phenotypic variation supports the hypothesis that both subspecies of *P. theca* deserves recognition at the species-level under the general lineage concept of species^4^,^34^.

Two neutrality tests were chosen for the demographic history analysis, and significantly negative values of neutrality statistics can be indicative of background selection, but are also consistent with either population subdivision or expansion. Fu’s Fs is significantly negative in *P. t. fukia* group reveal that the mtDNA of *P. t. fukia* appear to be not evolving neutrally. Additionally, we analysed the population size change of *P. t. theca, P. t. fukia* and all *P. theca*, respectively by the software DnaSP4.90^23^ and got no evidence for population expansion in *P. t. theca* group^31^ (Fig. 5A). However, we got unimodal curves representing population expansion in *P. t. fukia*^31^ (Fig. 5B). The results suggest that population expansion in *P. t. fukia*. We still confirm the result of the population size change in haplotype network (Fig. 3). Statistical parsimony network reflects genealogical relationships of the mtDNA haplotypes, that is, single mutation steps separate adjacent haplotypes in the network and older haplotypes are placed at internal branching points whereas younger ones occur toward the tip positions^35^. The haplotypes network of *P. t. fukia* displays a star-like pattern (Fig. 3). Haplotype I, the second most common and geographically widespread in central-west of China, is in the star’s centre and derivatives are connected to it by short branches. Based on coalescence theory, the star-like topologies for this cluster strongly suggest the effect of a population expansion^36^. In our study, a higher F_ST_ value indicated a lower level of gene flow (Nm) and higher genetic differentiation among populations. The results of two-level AMOVA show that significant genetic variation exists among the examined populations. These results provide a second line of support to a conclusion that the *P. t. fukia* is a different species.

In conclusion, our investigations and analyses revealed significant molecular and biogeographical differences between *P. t. theca* and *P. t. fukia*. We propose that *P. t. fukia* should be treated as a distinct species called *Polytremis fukia* Evans 1940 under the Phylogenetic Species Concept. In fact, it has been recently found that other species previously considered subspecies based on morphology are in fact sibling species that passed unnoticed until the advent of molecular techniques^37^,^38^,^39^,^40^. Results from our study strengthen information about the *Polytremis* species complex and help in developing appropriate integrated pest management strategies for these insect pests.

## Additional Information

**How to cite this article**: Jiang, W. *et al*. Taxonomic status and molecular phylogeography of two sibling species of *Polytremis* (Lepidoptera: Hesperiidae). *Sci. Rep.* **6**, 20820; doi: 10.1038/srep20820 (2016).

## Figures and Tables

**Table 1:** List of specimen information used for this study.

Locality	Specimen ID	Sex	Date	Altitude		Accession number	
					Haolotype (COI/*wingless*)	COI	*wingless*
*Polytremis theca theca*							
Lianglu, Tianquan County, Sichuan Province (N 29°55′; E 102°23′)	Ptt_LL 1	♀	6-Jul-11	not recorded	Ptt I/Ptt I	KC684410*	KR911938
	Ptt_LL 2	♂	11-Aug-11	not recorded	Ptt I/Ptt II	KC684410*	KR911939
Wolong, Wenchuan County, Sichuan Province (N 31°29′; E 103°35′)	Ptt_WL 1	♂	6-Aug-11	not recorded	Ptt II/Ptt II	KC684411*	KR911939
	Ptt_WL 2	♂	6-Aug-11	not recorded	Ptt II/Ptt II	KC684411*	KR911939
Luding, Sichuan Province (N 29°55′; E 102°15′)	Ptt_LD 1	♂	19-Jul-11	not recorded	Ptt II / Ptt II	KC684411*	KR911939
Feng County, Shanxi Province (N 33°54′; E 106°31′)	Ptt_FC 1	♀	19-Jul-11	not recorded	Ptt III/Ptt I	KR911924	KR911938
	Ptt_FC 2	♂	19-Jul-11	not recorded	Ptt III/Ptt I	KR911924	KR911938
Qinling, Shanxi Province 34°14′N 103°54′E	Ptt_QL 1	♂	16-Jul-10	not recorded	Ptt III/Ptt I	KR911924	KR911938
*Polytremis theca fukia*							
Qinliangfeng, Lin’an County, Zhejiang Province (N 30°59′; E 118°52′)	Ptf_QLF 1	♂	11-Apr-13	800 m	Ptf II/Ptf I	KC684406*	KR911935
Tianmushan, Lin’an County, Zhejiang Province (N 30°19′; E 119°25′)	Ptf_TMS 1	♀	8-May-09	not recorded	Ptf I/Ptf I	KC684409*	KR911935
	Ptf_TMS 2	♂	20-Sep-08	not recorded	Ptf I/Ptf I	KC684409*	KR911935
	Ptf_TMS 3	♂	17-Aug-09	not recorded	Ptf I/Ptf I	KC684409*	KR911935
	Ptf_TMS 4	♂	20-Aug-13	300 m	Ptf II/Ptf I	KC684406*	KR911935
Wuyishan, Fujian Province (N 27°44′; E 118°01′)	Ptf_WYS 1	♂	19-Sep-08	not recorded	Ptf III/Ptf II	KC684408*	KR911936
	Ptf_WYS 2	♂	19-Sep-08	not recorded	Ptf III/Ptf II	KC684408*	KR911936
Jingangshan, Ji’an county, Jiangxi Province (N 26°45′; E 114°17′)	Ptf_JGS 1	♀	19-Oct-10	not recorded	Ptf I/Ptf I	KC684409*	KR911935
	Ptf_JGS 2	♀	19-May-10	not recorded	Ptf II/Ptf I	KC684406*	KR911935
	Ptf_JGS 3	♀	20-May-10	not recorded	Ptf II/Ptf I	KC684406*	KR911935
Yueshan, Fengxin County, Jiangxi Province (N 28°79′; E 115°18′)	Ptf_YS 1	♀	13-Jul-13	400–600 m	Ptf II/Ptf I	KC684406*	KR911935
	Ptf_YS 2	♀	13-Jul-13	400–600 m	Ptf IV/Ptf I	KR911919	KR911935
Maoershan, Xin’an County, Guangxi Province (N 25°49′; E 110°00′)	Ptf_MES 1	♀	8-Jul-11	400 m	Ptf V/Ptf II	KC684407*	KR911936
	Ptf_MES 2	♂	7-Jul-11	400 m	Ptf II/Ptf III	KC684406*	KR911937
	Ptf_MES 3	♂	7-Jul-11	400 m	Ptf VI/Ptf II	KR911920	KR911936
	Ptf_MES 4	♀	7-Jul-11	400 m	Ptf VII / Ptf III	KR911921	KR911937
	Ptf_MES 5	♂	8-Jul-11	400 m	Ptf II/Ptf III	KC684406*	KR911937
	Ptf_MES 6	♂	8-Jul-11	400 m	Ptf II/Ptf III	KC684406*	KR911937
	Ptf_MES 7	♂	8-Jul-11	400 m	Ptf VIII/Ptf I	KR911922	KR911935
	Ptf_MES 8	♂	9-Jul-11	2000 m	Ptf VIII/Ptf I	KR911922	KR911935
Anjiangpin,Lingui County, Guangxi Province (N 25°14′; E 110°12′)	Ptf_AJP 1	♂	13-Jul-11	1200 m	Ptf I/Ptf III	KC684409*	KR911937
	Ptf_AJP 2	♂	13-Jul-11	1200 m	Ptf II/Ptf II	KC684406*	KR911936
	Ptf_AJP 3	♂	13-Jul-11	1200 m	Ptf IX/Ptf III	KR911923	KR911937
	Ptf_AJP 4	♂	14-Jul-11	1350 m	Ptf VIII/Ptf II	KR911922	KR911936
Bifengxia, Yaan County, Sichuan Province (N 30°03′; E 102°62′)	Ptf_BFX 1	♀	2-Aug-14	not recorded	Ptf II/Ptf I	KC684409*	KR911935
Outgroups							
*Polytremis nascens*							
Zhanghe, Langao County, Shaanxi Province (N 32°32′; E 108°14′)	Pn_ZH 1	♂	30-Jul-12	1600 m	Pn I/Pn I	KJ574014*	KR911931
	Pn_ZH 2	♂	30-Jul-12	1600m	Pn II/Pn I	KC684397*	KR911931
Hailuogou, Tianquan County, Sichuan Province (N 29°34′; E 102°04′)	Pn_HLG 1	♂	27-Jul-06	2300 m	Pn I/Pn II	KJ574014*	KR911932
	Pn_HLG 2	♂	27-Jul-06	2300 m	Pn II/Pn II	KC684397*	KR911932
Lianglu, Tianquan County, Sichuan Province (N 29°55′; E 102°23′)	Pn_LL 1	♂	3-Sep-10	1450 m	Pn I/Pn II	KJ574014*	KR911932
Fenghuangshan, Hanyin County, Shaanxi Province (N 32°90′; E 108°50′)	Pn_FHS 1	♀	3-Aug-12	1400 m	Pn II/Pn I	KC684397*	KR911931
	Pn_FHS 2	♀	3-Aug-12	1600 m	Pn III/Pn I	KJ574015*	KR911931
Houhe, Wufeng County, HuBei Province (N 30°22′; E 110°68′)	Pn_HH 1	♂	9-Jul-13	1200 m	Pn II/Pn III	KC684397*	KR911933
*Polytremis mencia*							
Tianmushan, Lin’an County, Zhejiang Province (N 30°19′; E 119°25′)	Pm_TMS 1	♂	5-Jun-11	not recorded	Pm I/Pm I	KC684415*	KR911940
	Pm_TMS 2	♂	15-Jun-10	not recorded	Pm I/Pm I	KC684415*	KR911940
	Pm_TMS 3	♂	30-May-10	not recorded	Pm II/Pm I	KC684414*	KR911940
	Pm_TMS 4	♀	20-Sep-08	not recorded	Pm III/Pm I	KC684416*	KR911940
Ningbo, Zhejiang Province (N 29°52′; E 121°32′)	Pm_NB 1	♂	23-Aug-11	not recorded	PmIV/Pm I	KC684417*	KR911940
*Polytremis pellucid*							
Tianmushan, Lin’an County, Zhejiang Province (N 30°19′; E 119°25′)	121112009	♀	19-Sep-08	not recorded	*P. pellucid*	KC684393*	KR911941
*Polytremis zina*							
Tianmushan, Lin’an County, Zhejiang Province (N 30°19′; E 119°25′)	121116021	♂	18-Aug-09	not recorded	*P. zina*	KC684395*	KR911942
*Polytremis discreta*							
Baoxing County, Sichuan Province (N 30°22′; E 102°48′)	121116024	♂	2-Jul-08	not recorded	*P. discreta*	KC684392*	KR911943
*Polytremis lubricans*							
Baoxing County, Sichuan Province (N 30°22′; E 102°48′)	121116027	♂	2-Jul-08	not recorded	*P. lubricans*	KC684391*	KR911944
*Polytremis eltola*							
Jingxiu, Guangxi Province (N 24°07′; E 110°11′)	121116030	♂	28-Jul-11	not recorded	*P. eltola*	KC684389*	KR911945
*Polytremis gigantean*							
Qingchengshan, Sichuan Province (N 30°53′; E 103°34′)	121119033	♀	28-Aug-11	not recorded	*P. gigantean*	KC684403*	KR911929
*Polytremis matsuii*							
Hongya, Sichuan Province (N 29°54′; E 103°22′)	121119036	♂	4-Jun-11	not recorded	*P. matsuii*	KC684400*	KR911927
*Polytremis caerulescens*							
Lianglu, Tianquan County, Sichuan Province (N 29°55′; E 102°23′)	121119039	♂	21-Jul-11	not recorded	*P. caerulescens*	KC684399*	KR911925
*Polytremis jigongi*							
Tianmushan, Lin’an County, Zhejiang Province (N 30°20′; E 119°23′)	121119042	♂	11-Jul-09	not recorded	*P. jigongi*	KC684404*	KR911934
*Polytremis kiraizana*							
Qilai, Taiwan Province (N 24°01′; E 121°22′)	121124051	♂	11-Jul-91	not recorded	*P. kiraizana*	KC684401*	KR911926
*Polytremis suprema*							
Jingxiu, Guangxi Province (N 24°07′; E 110°11′)	121124052	♂	31-Jul-11	not recorded	*P. suprema*	KC684402*	KR911928
*Polytremis gotama*							
Lijiang, Yunnan Province (N 26°51′; E 100°13′)	130101067	♂	28-Jul-06	not recorded	*P. gotama*	KC684396*	KR911930
*Borbo cinnara*							
Taidong, Taiwan Province (N 22°59′; E 120°59′)	121124069	♂	17-Sep-07	not recorded	*B. cinnara*	KC684418*	KR911947
*Pseudoborbo bevani*							
Nantou, Taiwan Province (N 23°55′; E 120°41′)	121124067	♀	14-Sep-03	not recorded	*P. bevani*	KC684419*	KR911946

^*^indicates the sequences retrieved from GenBank.

**Table 2:** Genetic diversity and neutrality tests calculated for *P. t. theca* and *P. t. fukia*.

	Ns	Nh	Hd	Nv	π	SD (π)	D	F
All *P. theca* samples (COI)	33	12	0.875	38	0.0207	0.0019	0.281	2.368
*P. t. theca* samples (COI)	8	3	0.750	6	0.0064	0.0048	1.598	2.631
*P. t. fukia* samples (COI)	25	9	0.803	13	0.0050	0.0070	–1.014	–1.886*
All *P. theca* samples (*wingless*)	33	5	0.773	9	0.0078	0.0057	1.122	1.509
*P. t. theca* samples (*wingless*)	8	2	0.571	1	0.0015	0.0010	1.444	1.100
*P. t. fukia* samples (*wingless*)	25	3	0.640	3	0.0030	0.0020	1.080	1.159

Ns–number of samples, Nh–Number of haplotypes, Hd–Haplotype diversity, Nv–Number of variable sites, π–nucleotide diversity, SD–standard deviation, D–Tajima’s D statistic, F–Fu’s F statistic, *–significant difference.

**Table 3:** Analysis of molecular variance (AMOVA) for the COI sequences of *P. t. theca* and *P. t. fukia.*

Source of variation	*df*	Sum of squares	Variance components	Percentage variation	Φ Statistic
Among populations	1	169.650	9.98266 Va	88.53	—
Within populations	11	45.269	1.29341 Vb	11.47	0.896 (p < 0.01)
Total	12	214.919	11.27607		
Fixation index	0.8853				

**Table 4:** Different morphological features of genitals and wings between *P. t. theca* and *P. t. fukia.*

	wing					genital
	Color of cilia of wings	Color of Underside ground	Number of spots in space Cu2 of the forewing	Color of scales scattered in costa and subapical area of forewing	Color of scales scattered in discal area and dorsum of hindwing	Ductus bursae
*P. t. theca*	brown	yellowish brown	0	greenish ochreous	greenish ochreous	thin
*P. t. fukia*	greyish white	greenish ochreous	1 or 2	greyish white	greyish white	thick
